# Experimental data of cement grouting in coarse soils with different superplasticisers

**DOI:** 10.1016/j.dib.2020.105612

**Published:** 2020-04-22

**Authors:** Costas A. Anagnostopoulos, Theodoros Chrysanidis, Maria Anagnostopoulou

**Affiliations:** Department of Environmental Engineering, International Hellenic University, 57400 Sindos, Thessaloniki, Greece

**Keywords:** Cement grouting, Superplasticisers, Shear strength, Triaxial test

## Abstract

High-range water reducers, such as superplasticisers, can be potentially viable for cement grouting applications. In this study, we investigate the influence of two types of superplasticisers—one based on polycarboxylate ether and another based on naphthalene condensates—on the injectability of thick cement grouts into coarse soil and on the shear strength parameters of the grouted soil. Injectability tests were performed on soil columns with various superplasticiser dosages and grouts prepared with different water-to-cement ratios over a wide range of grouting pressures. The shear strength parameters of the grouted soil were evaluated through undrained unconsolidated triaxial compression tests.

Specifications table

**Table:** 

Subject	Civil and Structural Engineering
Specific subject area	Construction Materials
Type of data	Tables, figures
How data were acquired	Injecting soil columns with cement grouts and applying triaxial compressive load on grouted soil samples.
Data format	Raw, calculated, analyzed, tabulated, plotted
Parameters for data collection	Data were obtained from injection experiments on soil columns with grouts proportioned with different superplasticiser types and dosages under a wide range of injection pressures.
Description of data collection	Injectability of grouts was measured in relation to the w/c ratio, superplasticiser type and dosage, and injection pressure. The shear strength parameters (friction angle and cohesion) of the grouted soil were obtained from undrained unconsolidated triaxial compression tests.
Data source location	Faculty of Environmental Engineering, International Hellenic University, Thessaloniki, Greece
Data accessibility	With the article

## Value of the Data

• We compare the injectability of superplasticised grouts based on polycarboxylate ether and polynaphthalene.
• We investigate the influence of the superplasticiser type on the shear strength of the grouted soil.
• This report will serve as a guideline for selecting appropriate parameters for superplasticised grouts, e.g., the type of cement and the combination of new-generation superplasticisers with other additives, in future research.
• Although laboratory simulation test results are not directly related to the actual physical filtration process of the grouts at the field scale, they provide valuable information on the flow behaviour of suspended cement particles in porous media during filtration, especially when combined with chemical admixtures, for estimating the results of in situ grouting.

## 1 Data Description

**Fig. 1:**
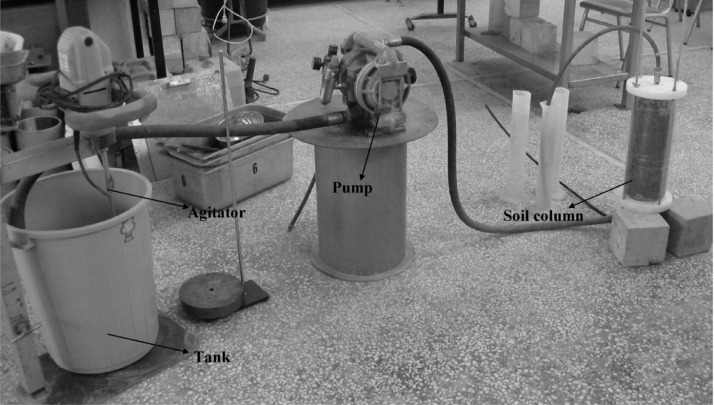
Setup for the injectability tests.

**Fig. 2:**
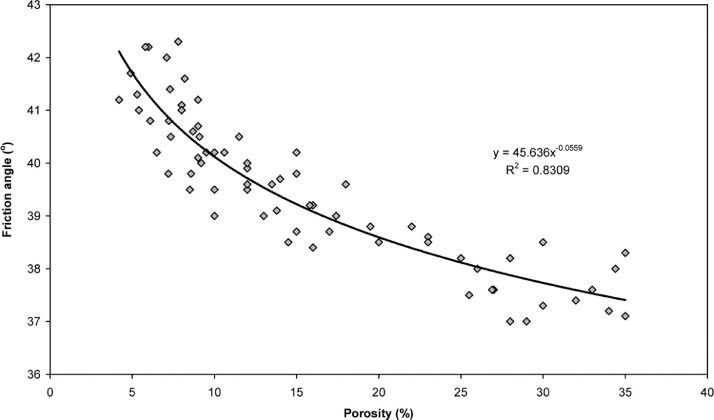
Friction angle of grouted specimens in relation to the porosity.

Table 1 presents the compositions of the superplasticised grouts used for the injection experiments in 150-cm-long soil columns. The average values of the friction angle and cohesion of the grouted soil columns are shown in Tables 2 and 3, respectively. A schematic of the experimental setup for the injectability tests is shown in Fig. 1. Table 4, Table 5, Table 6 summarize the injectability results of the different superplasticised grouts. Fig. 2 shows the relationship between the friction angle and the porosity of the grouted soil. More detailed information on the relationship of the cohesion and porosity of the grouted soil with the distance from the injection point can be found in the supplementary Excel datasets.

**Table 1:** Summary and notation of the injection experiments.

Notation	w/c	% SNF	% PCE	Injection pressure (bar)
G_1_	0.5	1	-	1
G_2_	0.5	1	-	6
G_3_	0.5	-	1	1
G_4_	0.5	-	1	6
G_5_	0.4	1.5	-	1
G_6_	0.4	1.5	-	6
G_7_	0.4	-	1.5	1
G_8_	0.4	-	1.5	6
G_9_	0.33	2.5	-	1
G_10_	0.33	2.5	-	6
G_11_	0.33	-	2.5	1
G_12_	0.33	-	2.5	6

**Table 2:** Average values of φ of grouted soil columns.

Notation	φ (degrees)	Difference (%)
G_1_	39.22	3.23
G_2_	39.42	3.75
G_3_	40.04	5.37
G_4_	40.28	6.01
G_5_	37.85	-0.37
G_6_	38.27	0.71
G_7_	40.97	7.8
G_8_	40.94	7.74
G_9_^a^	37.6	-1.05
G_10_	37.72	-0.71
G_11_^b^	39.8	4.73
G_12_	40.67	7.03

a It refers to the 1^st^ 20 cm-length grouted part.

b It refers to the 1^st^ 40 cm-length grouted part.

**Table 3:** Average values of c of grouted soil columns.

Notation	c (MPa)
G_1_	5.2
G_2_	6.75
G_3_	6.6
G_4_	7.66
G_5_	1.14
G_6_	2.78
G_7_	8.3
G_8_	9.51
G_9_^a^	0.84
G_10_	1.85
G_11_^b^	3.4
G_12_	11.8

a It refers to the 1^st^ 20 cm-length grouted part.

b It refers to the 1^st^ 40 cm-length grouted part.

**Table 4:** Penetration length or passing volume of PCE or SNF grouts with w/c ratio of 0.5, obtained from injectability tests.

Injection pressure (bar)	PCE grout		SNF grout		Superplasticiser dosage %
	Penetration length (cm)	Volume of grout passed (l)	Penetration length (cm)	Volume of grout passed (l)	
1	18	-	Negligible	-	0.5
2	25	-	Negligible	-	
3	35	-	Negligible	-	
4	-	2.4	8	-	
5	-	3.8	15.5	-	
6	-	4.3	22	-	
1	-	0.82	Negligible	-	0.75
2	-	2	Negligible	-	
3	-	4.5	7	-	
4	-	10	16	-	
5	-	All passed	25	-	
6	-	All passed	30	-	
1	-	1.6	-	0.09	1
2	-	4	-	0.12	
3	-	8.5	-	0.15	
4	-	All passed	-	0.18	
5	-	All passed	-	0.21	
6	-	All passed	-	0.25	
1	-	4	-	0.16	1.25
2	-	10	-	1.4	
3	-	22	-	3.5	
4	-	All passed	-	4.7	
5	-	All passed	-	6.5	
6	-	All passed	-	9

**Table 5:** Penetration length or passing volume of PCE or SNF grouts with w/c ratio of 0.4, obtained from injectability tests.

Injection pressure (bar)	PCE grout		SNF grout		Superplasticiser dosage %
	Penetration length (cm)	Volume of grout passed (l)	Penetration length (cm)	Volume of grout passed (l)	
1	9	-	Negligible	-	0.75
2	25	-	Negligible	-	
3	-	0.8	Negligible	-	
4	-	3	7	-	
5	-	9.5	13	-	
6	-	All passed	19	-	
1	33	-	5	-	1
2	-	1.1	11	-	
3	-	3.6	18	-	
4	-	10.5	26	-	
5	-	15	36	-	
6	-	All passed	-	0.05	
1	39	-	13	-	1.25
2	-	2.2	29	-	
3	-	6.4	-	0.4	
4	-	12.3	-	1.1	
5	-	18.8	-	1.9	
6	-	All passed	-	2.5	
1	-	0.2	28	-	1.5
2	-	7.5	-	1.3	
3	-	17	-	3.2	
4	-	All passed	-	5.8	
5	-	All passed	-	7.5	
6	-	All passed	-	10.5

**Table 6:** Penetration length or passing volume of PCE or SNF grouts with w/c ratio of 0.33, obtained from injectability tests.

Injection pressure (bar)	PCE grout		SNF grout		Superplasticiser dosage %
	Penetration length (cm)	Volume of grout passed (l)	Penetration length (cm)	Volume of grout passed (l)	
1	22	-	Negligible	-	1.25
2	-	1.1	Negligible	-	
3	-	3.5	Negligible	-	
4	-	6.2	36	-	
5	-	11.4	-	0.2	
6	-	All passed	-	0.8	
1	-	0.5	Negligible	-	1.75
2	-	4	19	-	
3	-	13	-	0.2	
4	-	25	-	2.3	
5	-	All passed	-	5.5	
6	-	All passed	-	8.2	
1	-	2.6	24	-	2.25
2	-	6.8	-	0.3	
3	-	16.5	-	2.1	
4	-	35	-	5.7	
5	-	All passed	-	7.8	
6	-	All passed	-	12.5	
1	-	3.8	32	-	2.75
2	-	10.8	-	1.9	
3	-	26.4	-	5.5	
4	-	All passed	-	12.5	
5	-	All passed	-	18.7	
6	-	All passed	-	24

## 2 Experimental Design, Materials and methods

### 2.1 Materials

The experiments were performed using a common type of Portland cement (CEM II/B-M 42.5 N). A polycarboxylate ether superplasticiser (PCE) acting by steric forces and a polynaphthalene-based superplasticiser (SNF) acting by electrostatic forces were selected as additives [1]. Coarse soil of poorly graded limestone with a relative density, D_r_, of 95% was used in the injection tests. Its grain size ranged between 4.76 and 2.38 mm, with a uniformity coefficient of 1.6, dry unit weight of 14.8 kN/m^3^, void ratio of 0.75, porosity of 43%, and friction angle, φ, of 38°. The φ value of the coarse soil was obtained from undrained unconsolidated (UU) direct shear tests conducted on dry specimens under normal stresses of 100, 200, 400, and 800 kPa

### 2.2 Methods

To determine the shear strength parameters of the grouted soil and their relation to factors such as the water-to-cement ratio (w/c), superplasticiser type, and injection pressure, injection experiments were carried out on soil columns at constant pressures of 1 and 6 bar with grouts having a w/c ratio of 0.5, 0.4, and 0.33. A summary of the grouting experiments and the notations used are presented in Table 1. The apparatus used for the grouting process was constructed according to the ASTM D4320-04 specification. The soil columns were made of a plastic tube with a height of 1500 mm and an internal diameter of 94 mm, and were filled with dry, dense coarse soil (D_r_ of 95%) [2]. The grout was blended using a high-speed paddle mixer with three blades according to the ASTM C938-10 specification. A high shear blending system was employed to ensure the total dispersion of the cement particles. The delayed-addition method was used to prepare the superplasticised grouts. Specifically, the required amounts of cement and water were blended for 5 min, and, after 2 min of static hydration, the appropriate amount of superplasticiser was added. Then, the resulting mixture was blended for at least 2 min to achieve a uniform dispersion of the cement particles [3]. The grouts were continuously agitated during the injection experiments and were injected from the bottom of the soil column. The grouting process was stopped when no flow of grout from the upper outlet hose of the soil column was observed or when there was no indication that grout was flowing through the soil specimen. The grouted specimens were left on their base for three days in a vertical position and then demoulded and stored until the day of testing. After 30 days of curing, the grouted columns were cut into 20-cm-long segments. From each segment, a core sample with a diameter of 6.3 cm and a height of 12.5 cm was extracted by using a drilling machine. These core samples were tested under UU triaxial compression for the estimation of the shear strength parameters, i.e., φ and the cohesion, c. The UU triaxial tests were performed under constant confining pressures of 0.5, 1.0, and 2.0 MPa. The description of the triaxial testing apparatus used in the experiments is presented in a previous study [4].

The injectability of the grouts containing different dosages of superplasticiser was evaluated by injecting dry, dense, and coarse soil (D_r_ of 95%) into columns with a diameter of 14.4 cm and a height of 40 cm. A transparent tube was utilised for the formation of the soil columns, to facilitate visual observations of the flow of the grouts (Fig. 1). The pressure applied during the injection experiments varied between 1 and 6 bar. For each value of the injection pressure, the total volume of the grout that passed through when the grout stopped flowing or when the maximum penetration distance of the grout inside the soil column was recorded. The maximum penetration distance of the grout was estimated visually by averaging three measurements on different sides of the column. The quantity of the grout that passed through the specimen was measured using graduate cylinders. To ensure reliable test results, the volume of each of the reported grouts is taken as the average value of at least three measurements, with a maximum allowable deviation of 1% from the average.

The porosity of the grouted soil was estimated from specimens with the same size as the one for the mechanical tests and aged for 30 days using the vacuum saturation technique according to the ASTM C 1202-19 specification.

### 2.3 Effect of superplasticiser type on the shear strength parameters

The shear strength parameters of the grouted soil columns were determined from a total stress analysis for plotting the modified failure envelope in p-q diagrams. The failure envelopes for all cases appeared to be linearly proportional to the correlation coefficients R^2^ ranging from 0.98 to 0.99, which indicates the suitability of the Mohr–Coulomb failure criterion as an indicator of the mechanical behaviour of the grouted soil. Tables 2 and 3 list the obtained average values of φ and c of the grouted soil columns. The triaxial test results showed that shear strength increment was directly related with the development of a significant c, while the increase in φ was limited. The specimens grouted with PCE grouts developed higher c than those grouted with SNF grouts. In the PCE grouted specimens, lower w/c ratios led to greater c. In contrast, in the SNF grout specimens, lower w/c ratios resulted in significantly lower c. Moreover, the injection pressure affected c with SNF grouts more than with PCE grouts.

In most of the cases, the grouted soils exhibited limited φ increments, and in a few grouted soil samples with SNF grouts, φ appeared to be slightly lower than that of the ungrouted soil. In general, φ decreased with increasing distance from the injection point, which can be attributed to the porosity of the grouted soil. Fig. 2 shows the relation between φ and the porosity. From the figure, it can be seen that φ decreases with increasing porosity; although there is some variation, a clear power law can be observed. Most likely, the produced cementitious material not only binds the soil grains, contributing to the overall increase in matrix cohesion, but also fills the pore space and imparts friction by restricting the relative movement between soil grains during shearing. This explains why PCE grouts resulted in a greater increase in φ than the SNF grouts; the filling of voids achieved with PCE grouts was higher.

### 2.4 Effect of superplasticiser type on injectability

All the injection tests performed in this study indicated that the injectability of the grouts is strongly dependent on the type and dosage of superplasticiser, grouting pressure, and w/c ratio. Table 4, Table 5, Table 6 summarise the penetration length and the volume of the grouts with different compositions that passed through the soil columns in relation to the aforementioned parameters. The injectability of PCE grouts was observed to be greater than that of the SNF grouts. Particularly, under high pressure, all of the PCE grouts (50 l) in the tank passed through the soil, regardless of the w/c ratio; this did not occur for SNF grouts, even in the case of high doses. Furthermore, under low applied pressures, the penetration of SNF grouts was negligible compared to that of the PCE grouts.

## Conflict of Interest

The authors declare that they have no known competing financial interests or personal relationships that could have appeared to influence the work reported in this paper.

## Appendix Supplementary materials

Image, application 1

Image, application 2

Image, application 3
